# Oxylipins in triglyceride-rich lipoproteins of dyslipidemic subjects promote endothelial inflammation following a high fat meal

**DOI:** 10.1038/s41598-019-45005-5

**Published:** 2019-06-17

**Authors:** Anita Rajamani, Kamil Borkowski, Samir Akre, Andrea Fernandez, John W. Newman, Scott I. Simon, Anthony G. Passerini

**Affiliations:** 1 Department of Biomedical Engineering, University of California, Davis, 451 Health Sciences Dr., Davis, CA 95616 USA; 2 West Coast Metabolomics Center, Genome Center, University of California, Davis, 451 Health Sciences Dr., Davis, CA 95616 USA; 3 Department of Nutrition, University of California, Davis, 3135 Meyer Hall, One Shields Avenue, Davis, CA 95616 USA; 4 Western Human Nutrition Research Center, Obesity and Metabolism Research Unit, Agricultural Research Service, United States Department of Agriculture, 430 West Health Sciences Dr., Davis, CA 95616 USA

**Keywords:** Lipoproteins, Mechanisms of disease

## Abstract

Elevated triglyceride-rich lipoproteins (TGRL) in circulation is a risk factor for atherosclerosis. TGRL from subjects consuming a high saturated fat test meal elicited a variable inflammatory response in TNFα-stimulated endothelial cells (EC) that correlated strongly with the polyunsaturated fatty acid (PUFA) content. This study investigates how the relative abundance of oxygenated metabolites of PUFA, oxylipins, is altered in TGRL postprandially, and how these changes promote endothelial inflammation. Human aortic EC were stimulated with TNFα and treated with TGRL, isolated from subjects’ plasma at fasting and 3.5 hrs postprandial to a test meal high in saturated fat. Endothelial VCAM-1 surface expression stimulated by TNFα provided a readout for atherogenic inflammation. Concentrations of esterified and non-esterified fatty acids and oxylipins in TGRL were quantified by mass spectrometry. Dyslipidemic subjects produced TGRL that increased endothelial VCAM-1 expression by ≥35%, and exhibited impaired fasting lipogenesis activity and a shift in soluble epoxide hydrolase and lipoxygenase activity. Pro-atherogenic TGRL were enriched in eicosapentaenoic acid metabolites and depleted in esterified C18-PUFA-derived diols. Abundance of these metabolites was strongly predictive of VCAM-1 expression. We conclude the altered metabolism in dyslipidemic subjects produces TGRL with a unique oxylipin signature that promotes a pro-atherogenic endothelial phenotype.

## Introduction

Disorders of metabolism are accompanied by an elevated systemic inflammatory state that accelerates the progression of atherosclerotic cardiovascular disease (CVD), the leading cause of death and disability in our society^1,2^. One-third of Americans are clinically categorized at higher risk for CVD in that they show a cluster of risk factors associated with high fat western diet and sedentary lifestyle defined as metabolic syndrome^3^. Among these risk factors is hypertriglyceridemia, which is characterized by elevated levels of serum triglycerides, circulating in ApoB-containing triglyceride-rich lipoprotein (TGRL). Elevated TGRL is a strong predictor for CVD^4–7^, and its uptake by the endothelium leads to inflammation that precedes and promotes atherosclerosis. Here we investigate how changes in the relative abundance of fatty acid metabolites circulating in TGRL link to its ability to prime an inflammatory response to a high fat meal.

Atherosclerosis has been proposed to be a postprandial process that is tied to transient elevation of postprandial triglycerides^8,9^. A high fat meal induces a postprandial inflammatory state, characterized by elevated levels of circulating TGRL and other inflammatory mediators, including cytokines (e.g. TNFα) that promote endothelial dysfunction accompanying atherosclerosis. A high-fat test meal challenge has been established as an effective tool to elicit inflammation of arterial endothelium and subsequent monocyte capture, changes not typically observed under fasting conditions. Therefore, a postprandial response may provide greater insight into the effects of metabolism on insipient inflammation than the fasting state^10,11^. We have investigated the capacity of TGRL produced by human subjects after consuming a high saturated fat meal, typical of western diet, to elicit a pro-atherogenic endothelial response.

Among the earliest inflammatory responses by the endothelium that precedes atherosclerosis is the acute upregulation of cell adhesion molecules (CAMs), notably vascular cell adhesion molecule (VCAM-1), the predominant receptor for monocyte recruitment from the circulation^4,12^. VCAM-1 expression correlates with coronary lesion severity in atherosclerotic patients, and is causative in mouse models of atherosclerosis^13,14^. We showed that TGRL isolated from subjects after a standardized fast food breakfast meal high in saturated fat, can prime either a pro- or anti-atherogenic state, defined as a net up- or down-regulation of VCAM-1 expression in TNFα-stimulated human aortic endothelial cells (HAEC)^15–18^. In these studies, the level of VCAM-1 expression was strongly associated with the relative capacity to induce recruitment of monocytes from healthy subjects assessed in TNFα-inflamed HAEC^16^. The capacity for TGRL to alter VCAM-1 expression correlated closely with an individual’s metabolic status as reflected by their level of postprandial triglycerides and abdominal obesity. However, TGRL obtained from the same individuals when fasting did not prime inflamed HAEC for increased VCAM-1 expression^15^. Unknown is how the composition of TGRL is altered postprandially following the high fat meal to elicit a distinct endothelial inflammatory response by individuals exhibiting metabolic risk factors.

Dietary fatty acids of all saturation levels are packaged into and circulated by TGRL, which consist of chylomicrons and VLDL, synthesized in the gut and liver respectively^8^. The fatty acid composition of circulating TGRL thus reflects both the content of a meal and an individual’s overall metabolic state, including the presence of diabetes or dyslipidemia^19^. We previously reported that pro- and anti-atherogenic TGRL were composed of distinct fatty acid profiles, and that pro-atherogenic TGRL in particular was enriched in non-esterified polyunsaturated fatty acids (PUFA)^16^. Though overall less abundant in TGRL, PUFA and their oxygenated metabolites, *oxylipins*, are potent mediators of inflammation^20^. PUFA are metabolized by three main enzymatic pathways to produce an array of oxylipins: cyclooxygenases (COX), lipoxygenases (LOX), and cytochrome P450s (CYP). COX-derived prostaglandins are generally considered pro-inflammatory. While LOX-derived mid-chain alcohols, leukotrienes, and ketones can be either pro- or anti-inflammatory (depending on the LOX isoform, the parent fatty acid and hydroxylation position), CYP-derived epoxides are considered anti-inflammatory. However, epoxides are readily hydrolyzed by soluble epoxide hydrolase (sEH) to form diols, which are reported to promote or have a neutral effect on inflammation^20^. Fatty acids and oxylipins are found in both esterified (bound) and non-esterified (free) pools in TGRL, and can be released by the action of lipases or upon receptor-mediated uptake by endothelial cells (EC)^21^.

Although 90% of oxylipins are esterified in lipoproteins, their exact origins are unclear, and the significance of their transport within lipoproteins is largely unknown. It has been demonstrated that diet and metabolism affect oxylipin abundance and distribution among lipoprotein pools. For example, plasma oxylipins were enriched in proportion to the fatty acid composition of a high-fat meal^10,11^. Metabolic syndrome is associated with a disruption of the oxylipin pattern across all lipoprotein classes that was putatively pro-inflammatory, and was mitigated by prescription omega-3 PUFA intervention^22^. Moreover, there is evidence that oxylipin metabolism can directly affect atherosclerosis. For example, inhibition of sEH reduced atherosclerosis in ApoE null mice on a high fat diet, which correlated with enrichment in the plasma epoxide to diol ratio^23^. We address whether oxylipins circulating in TGRL act to exacerbate endothelial inflammatory responses promoting atherosclerosis.

The objective of the current study was to identify, using an unbiased metabolomics approach, novel fatty acid constituents that can significantly predict the pro- or anti-atherogenic postprandial response after uptake and processing of an individual’s TGRL by endothelium. We hypothesized that metabolic dysregulation in dyslipidemic individuals is associated with a unique fasting and postprandial TGRL oxylipin signature in response to a high fat test meal. We report that the composition of sEH-derived diols and LOX-derived alcohols in postprandial TGRL strongly discriminated the response to the meal between pro- and anti-atherogenic subjects. A postprandial oxylipin signature was identified in pro-atherogenic subjects, characterized by enrichment in eicosapentaenoic acid (EPA) metabolites and depletion of esterified sEH-derived diols. Moreover, fasting indices of lipogenesis activity and the abundance of sEH-derived diols in TGRL were the best predictors of enhanced VCAM-1 expression.

## Results

### Plasma markers of dyslipidemia correlate with TGRL priming of HAEC inflammation

**Figure 1:**
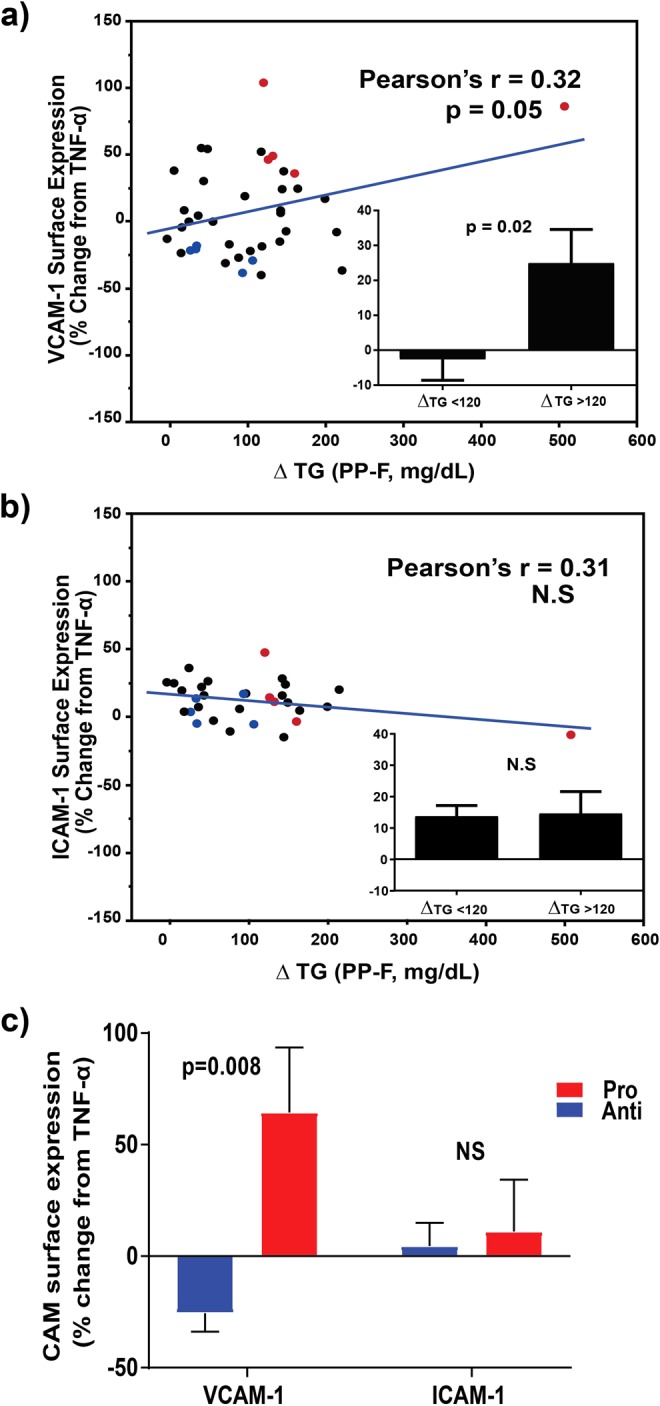
Postprandial TGRL modulates VCAM-1 surface expression in endothelial cells in proportion to an individual’s postprandial change in triglycerides. Human aortic endothelial cells (HAEC) were treated with TNFɑ (0.3 ng/ml) in the presence or absence of postprandial TGRL (10 mg/dl ApoB) for 4 hr, and CAM surface expression quantified by flow cytometry and reported relative to TNFα stimulation alone. (**a**) VCAM-1 expression across all subjects (black, N = 39) significantly correlated with the change in serum triglycerides in response to the meal. A threshold for change in plasma triglycerides (ΔTG, postprandial - fasting) >120 mg/dl distinguished the capacity of a subject’s TGRL to elicit an increase in VCAM-1 surface expression (inset). (**b**) There was no such correlation with ICAM-1 expression. (**c**) Postprandial TGRL from pro-atherogenic subjects (red, n = 5) significantly enhanced VCAM-1 expression compared to anti-atherogenic subjects (blue, n = 5), but did not differ with respect to their effect on ICAM-1 expression.

To evaluate the capacity of TGRL to mediate an inflammatory response in arterial endothelium, we examined a cohort of 39 subjects, characterizing each individual by their anthropometric, lipid, and metabolic characteristics, fasting and 3.5 hrs after the high fat test meal (Supplementary Table S1). Subjects exhibited a broad range in BMI (19.81–30.25 kg/m^2^) and waist circumference (24.5–43.0 in), and ranged from normal to hypertriglyceridemic (fasting triglycerides 30–279 mg/dl). We measured the capacity of postprandial TGRL isolated from each subject’s plasma to alter TNFα-stimulated CAM surface expression in HAEC in an *ex vivo* assay. An increase in VCAM-1 surface expression from stimulation with a low dose of TNFα (EC_50_ = 0.3 ng/ml) correlated most strongly with the postprandial spike in triglyceride levels (ΔTG = postprandial - fasting) (Fig. 1). Notably, postprandial TGRL isolated from subjects whose triglycerides increased >120 mg/dl in response to the meal routinely increased TNFα-stimulated VCAM-1 surface expression. In contrast, postprandial TGRL from those subjects in whom triglycerides increased <120 mg/dl reduced or had a neutral effect on TNFα-stimulated VCAM-1 surface expression in HAEC (Fig. 1a, inset). Each increase in plasma triglycerides of 100 mg/dl corresponded to a 12.5% increase in TNFα-stimulated VCAM-1 expression. In contrast, the change in triglycerides did not alter the inflammatory upregulation of ICAM-1 expression across subjects, which tended to be less variable (Fig. 1b, inset).

In order to evaluate changes in the composition of TGRL that correlate most strongly with a characteristic postprandial inflammatory response in HAEC, five individuals were selected for metabolomics analysis from this cohort whose TGRL produced a *pro-atherogenic* (pro-) response, eliciting the greatest increase in VCAM-1 surface expression (47.4 ± 22.5%) and expressing a ΔTG > 120 mg/dl. Another five subjects were selected whose TGRL produced an *anti-atherogenic* (anti-) response, eliciting the greatest decrease in VCAM-1 surface expression (40.2 ± 19.5%) and expressing a ΔTG < 120 mg/dl (Fig. 1c). The subject characteristics and response to the meal are reported in aggregate (Supplementary Table S2) for comparison to the larger study cohort (Supplementary Table S1). On average triglycerides more than doubled in these subjects after the meal. Consistent with the larger study cohort all subjects exhibited substantially increased TG:HDL ratio and TC:HDL ratio 3.5 hrs postprandial compared to fasting. Postprandial TGRL did not significantly alter the TNFα-stimulated increase in ICAM-1 surface expression, which is consistent with previous studies^16,17^ (Fig. 1c).

We next compared anthropometric characteristics, lipid, and metabolic profiles between these same 10 subjects stratified into pro- and anti-atherogenic responders (Table 1). Consistent with our previous studies, pro-atherogenic subjects were distinguished by markers of abdominal obesity (higher BMI and waist circumference), and enrichment in ApoB containing lipoproteins (notably triglycerides), accompanied by lower HDL, both fasting and postprandial. Subjects did not meet the clinical criteria for metabolic syndrome^24^ since waist circumference, fasting glucose, and blood pressure were within normal ranges. The modulation of TNFα-stimulated VCAM-1 surface expression by postprandial TGRL in these subjects correlated directly with fasting triglycerides, TG:HDL, ApoB, TC:HDL, and inversely correlated with HDL (Table 2). This is noteworthy since TGRL isolated from subjects after an overnight fast did not itself alter endothelial inflammation. Therefore, in the following investigation into the effect of the meal on pro- and anti-atherogenic TGRL composition, oxylipin and fatty acid abundance are expressed as a ratio of postprandial/ fasting. In contrast to VCAM-1, changes in ICAM-1 surface expression did not correlate with any clinical indicators of dyslipidemia.

**Table 1:** Anthropometric, lipid, and metabolic characteristics of subjects selected for metabolomics profiling stratified into anti- and pro- atherogenic responders.

Physical, Non-metabolic	Anti-atherogenic	Pro-atherogenic	p-value
Age (yrs)	47.4 ± 22.51	40.2 ± 19.51	0.52
BMI (kg/m^2^)	23.66 ± 2.57	30.5 ± 4.37	0.02
Waist circumference (cm)	86.11 ± 12.42	93.35 ± 8.26	0.02
Hip circumference (cm)	89.28 ± 5.13	101.35 ± 5.13	0.14
WHR	0.96 ± 0.02	0.92 ± 0.05	0.09
Systolic (mmHg)	116.2 ± 5.02	124.4 ± 8.68	0.1
Diastolic (mmHg)	71.2 ± 9.23	77.6 ± 12.46	0.21
**Metabolic**, **Non-lipid**			
Glucose- fasting (mg/dl)	88.2 ± 6.06	85.2 ± 6.83	0.6
Glucose- postprandial (mg/dl)	82.6 ± 6.95	75.2 ± 13.08	0.3
**Metabolic**, **Lipid**			
**Fasting**			
HDL (mg/dl)	63.4 ± 14.31	42.4 ± 12.4	0.06
LDL (mg/dl)^1^	109 ± 33.89	198 ± 157.77	0.14
TC:HDL	2.94 ± 0.36	6.56 ± 2.68	0.01
TG (mg/dl)	64.4 ± 11.08	191.4 ± 79.69	0.01
ApoB (mg/dl)	71.2 ± 15.35	144 ± 90.71	0.01
Non-HDL-C (mg/dl)	122 ± 34.45	236.4 ± 154.41	0.04
TC (mg/dl)	185.4 ± 46.32	278.8 ± 160.24	0.21
TG:HDL	1.08 ± 0.43	4.93 ± 3.1	0.01
**Postprandial**			
HDL (mg/dl)	63.2 ± 14.79	39.6 ± 10.09	0.05
TC:HDL-C	2.96 ± 0.37	6.86 ± 2.84^#^	0.01
TG (mg/dl)	122.8 ± 32.15^#^	400.4 ± 223.29^#^	0.01
ApoB (mg/dl)	74.83 ± 21.83	148.4 ± 86.6	0.02
Non-HDL-C (mg/dl)	124.6 ± 39.01	236.2 ± 159.35	0.1
TC (mg/dl)	187.8 ± 52.08	275.8 ± 164.97	0.25
TG:HDL	2.04 ± 1.34^#^	10.32 ± 1.34^#^	0.01

Mean ± SD, n = 5 per group. P-values for pro- vs. anti- comparison performed using Wilcoxon signed rank test. ^1^Postprandial LDL levels could not be calculated for the majority of subjects due to elevated triglycerides. ^**#**^Significant change postprandial to fasting within group.

**Table 2:** Correlation of endothelial VCAM-1 surface expression in response to postprandial TGRL with subject (n = 10) anthropometric characteristics and fasting measures of lipid metabolism.

Factor	Spearman’s ρ	P-value (two tailed)
**Physical**, **non-metabolic**		
Hip circumference (cm)	0.68	0.03
Body mass index (kg/m^2^)	0.75	0.01
Systolic (mmHg)	0.63	0.05
**Measures of dyslipidemia**		
Triglycerides (mg/dl)	0.87	0.001
TG:HDL	0.84	0.002
ApoB (mg/dl)	0.77	0.01
TC:HDL	0.75	0.01
HDL (mg/dl)	−0.65	0.04

### A high fat meal challenge exacerbates a postprandial inflammatory state in pro-atherogenic subjects

To further characterize the differences in postprandial inflammatory state in dyslipidemic subjects eliciting a pro-atherogenic response, a panel consisting of cytokines and other markers of systemic inflammation was measured in plasma. Postprandial plasma levels of CRP, IFNγ, s-VCAM-1, and IL-6 were all significantly elevated in concert with a subject’s TGRL-dependent rise in endothelial VCAM-1 surface expression (Table 3). Among the inflammatory markers, an increase in CRP levels in response to the meal was most indicative of the pro-atherogenic endothelial response (Spearman’s ρ = 0.7, p = 0.03). Elevated CRP levels in both the fasting and postprandial states strongly discriminated between pro- and anti-atherogenic subjects (Supplementary Fig. S1), consistent with its utility as a general biomarker of inflammation and cardiovascular risk^25^. Moreover, elevated CRP levels of this magnitude in metabolic syndrome were associated with increased liver enzyme activity^26,27^. Also notable was that the postprandial change in s-VCAM-1, but not s-ICAM-1, in plasma correlated directly with a subject’s TGRL-mediated atherogenic phenotype in the *ex vivo* assay, in which pro-TGRL enhanced endothelial VCAM-1, but not ICAM-1 surface expression (Fig. 1c). Together these data confirm that VCAM-1 upregulation in HAEC and in the circulating plasma are faithful inflammatory biomarkers that correlate with clinical measurers of dyslipidemia and metabolic dysregulation in the subject cohort.

**Table 3:** Correlation of endothelial VCAM-1 surface expression in response to postprandial TGRL with fold increase in abundance (postprandial/ fasting) of subject (n = 10) cytokine and inflammatory biomarker levels.

Inflammation marker	Spearman’s ρ	P-value
CRP	0.70	0.03*
IFN-γ	0.66	0.04*
s-VCAM-1	0.62	0.05*
IL-6	0.61	0.06
IL-10	0.32	0.37
s-ICAM-1	0.26	0.47
IL-1β	0.20	0.58
IL-17A	0.15	0.68
TNFα	−0.01	0.99

IL-1α levels were undetectable.

^*^Significant difference.

### A meal in high saturated fat increases n-6 oxylipins in circulating TGRL

**Figure 2:**
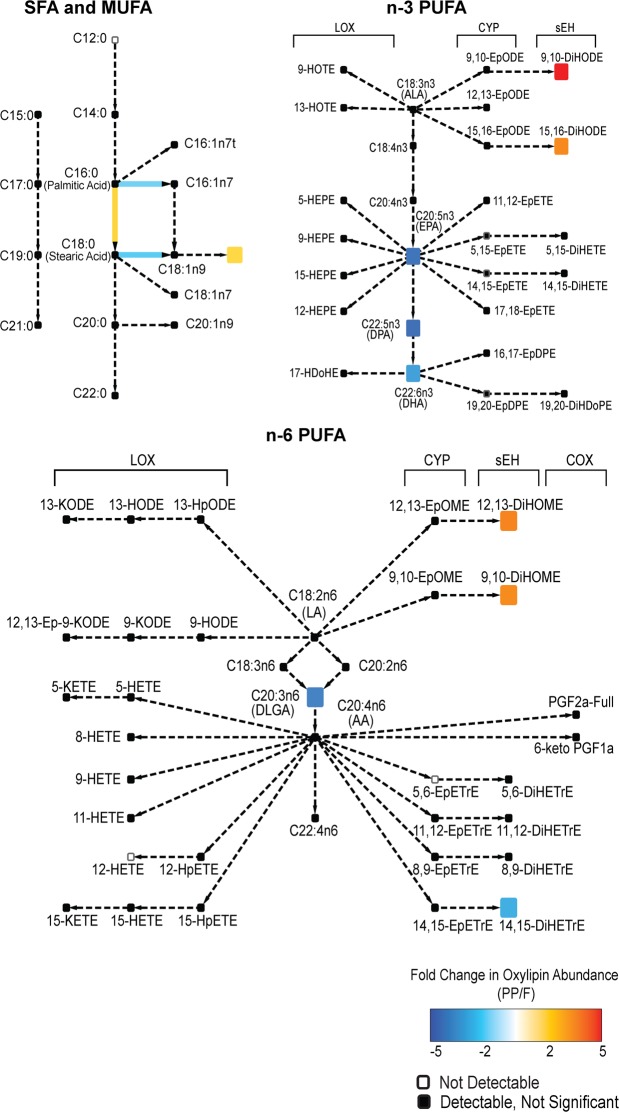
Network map depicting the fatty acids and metabolites measured in the esterified pool of TGRL. Superimposed in color are the fold change for fatty acids and oxylipins identified as significantly enriched (red) or depleted (blue) postprandially over all subjects. Colored arrows depict significant changes in the ratio of the abundance of the metabolites connected. Black boxes indicate metabolites that were detected but unchanged by the meal. White boxes are metabolites that were part of the analysis but not detected. Significance was determined using a two factor ANOVA comparing fasting to postprandial in the same subjects, and accounting for pro- and anti-atherogenic response. A Benjamini-Hochberg FDR multiple test correction was applied.

**Figure 3:**
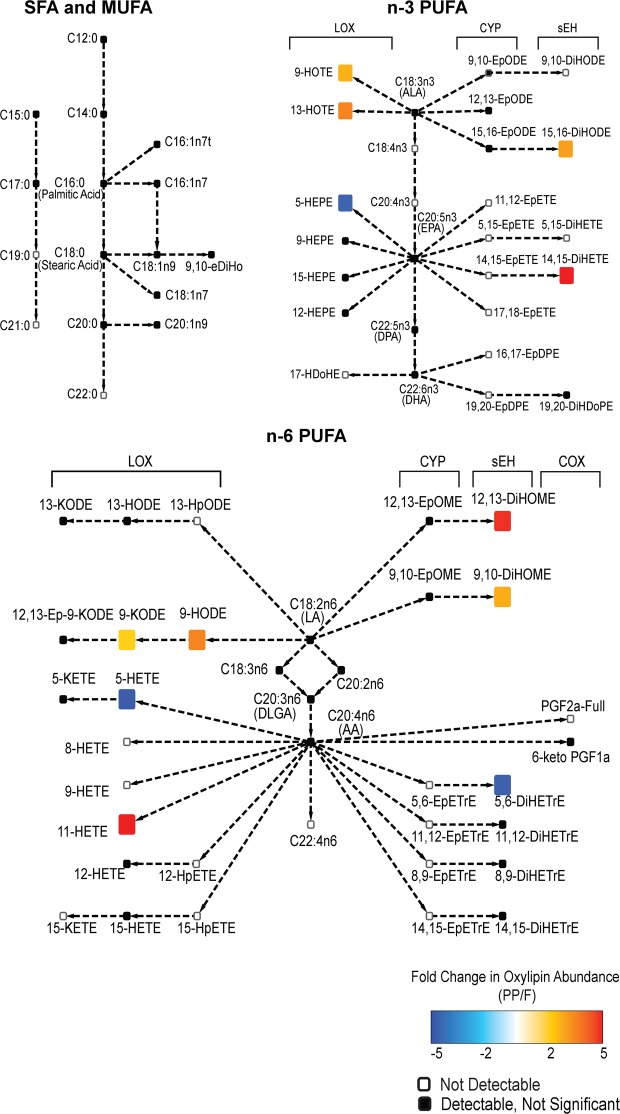
Network map depicting the fatty acids and metabolites measured in the non-esterified pool of TGRL. Superimposed in color are the fold change for fatty acids and oxylipins identified as significantly enriched (red) or depleted (blue) postprandially over all subjects. Colored arrows depict significant changes in the ratio of the abundance of the metabolites connected. Black boxes indicate metabolites that were detected but unchanged by the meal. White boxes are metabolites that were part of the analysis but not detected. Significance was determined using a two factor ANOVA comparing fasting to postprandial in the same subjects, and accounting for pro- and anti-atherogenic response. A Benjamini-Hochberg FDR multiple test correction was applied.

We next investigated whether the high fat test meal elicited an effect on specific classes of fatty acids or oxylipins across all subjects. The complete composition of the test meal is found in the supplement (Supplementary Table S3). A previous analysis of the postprandial composition of TGRL that was limited to parent fatty acids, revealed enrichment in PUFA in pro-TGRL^16^. Since PUFA are readily metabolized, this motivated a more detailed unbiased mass spectrometry analysis of the abundance of PUFA-derived oxylipins in addition to PUFA, SFA, and MUFA in fasting and postprandial TGRL. We identified 22 of 153 metabolites that changed significantly in response to the meal. The results are reported separately for the esterified and non-esterified pools, superimposed on network maps displaying the enzymes and parent fatty acids specific to each metabolite (Figs 2 and 3). Among the SFA, the test meal substantially increased the ratio of stearic (C18:0) to palmitic acid (C16:0) (Fig. 2), reflecting the higher abundance of stearic acid in the meal. An overload of SFA in response to the test meal was also evident as a decrease in the ratio of MUFA to SFA, particularly MUFAs derived from palmitic and stearic acids. In the esterified PUFA categories, there was significant postprandial reduction in abundance of several n-3 PUFAs, particularly docosahexaenoic acid (DHA), docosapentaenoic acid (DPA), and eicosapentaenoic acid (EPA), and in n-6 PUFA, dihomo-γ-linolenic acid (DGLA). There was a remarkable increase in abundance of sEH-derived oxylipins in response to the meal in both the esterified and non-esterified pools (Figs 2 and 3). Specifically, 12,13-dihydroxy octadecaenoic acid (12,13-DiHOME), 9,10-dihydroxy octadecaenoic acid (9,10-DiHOME), and 15,16-dihydroxy octadecadienoic acid (15,16-DiHODE) postprandially increased in abundance in both the esterified and non-esterified pools. The changes in the composition of the non-esterified pool were predominantly associated with sEH- and LOX-derived oxylipins originating from the C18 PUFA, linoleic (LA) and alpha-linolenic acid (ALA) (Fig. 3). These results highlight characteristic changes in the composition of TGRL after a meal high in saturated fat, including enrichment in diols and mid-chain alcohols that may mediate inflammation.

### sEH-derived diols and LOX-derived alcohols discriminate the response to the meal between pro- and anti-atherogenic subjects

**Figure 4:**
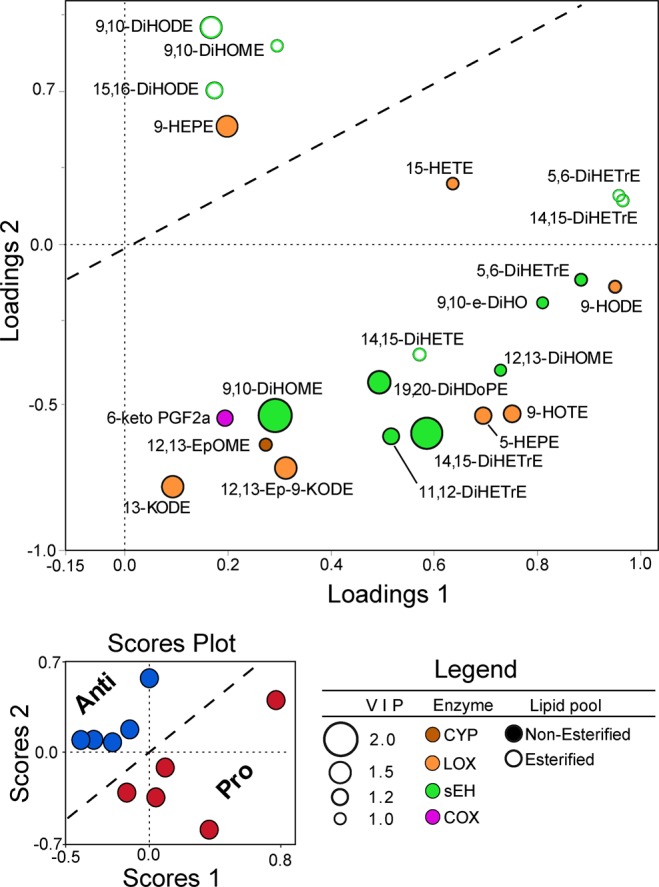
sEH-derived diols and LOX-derived alcohols strongly discriminate the response to the meal between pro- and anti-atherogenic subjects. PLS-DA analysis of postprandial TGRL samples characterized as pro- and anti-atherogenic (5 per group), using postprandial oxylipin abundance adjusted by fasting levels in the same individuals (postprandial/ fasting). The scores plot reveals the separation between pro- and anti-atherogenic-TGRL  (Q^2^ = 0.32). The PLS-DA model was constructed using all detected oxylipins. The loadings plot displays the 22 oxylipins that contributed significantly to discrimination between the groups (VIP > 1). The size of the circles corresponds to the VIP score. Each oxylipin is color coded by the enzyme from which it is derived.

To identify the differences in relative abundance of fatty acids and oxylipins in pro- vs. anti-atherogenic TGRL in response to the meal, a multivariate partial least squares discriminant analysis (PLS-DA) was performed. Postprandial levels of each constituent were normalized by fasting levels for each individual (postprandial/ fasting) to adjust for baseline metabolic variation. Oxylipins yielded the strongest discrimination between the pro- and anti-TGRL samples (Q^2^ = 0.32) (Fig. 4). In contrast, discrimination was not achieved with the addition of the abundance of parent fatty acids to the analysis. The scores plot displays the separation between the pro- and anti-atherogenic TGRL groups, and the loadings plot superimposes the 22 oxylipins that contributed significantly to the separation. Differential enrichment in the free to bound oxylipin pools (indicated by solid versus open circles) emerged as a major trend distinguishing pro-TGRL. Mapping to the enzymatic pathways, distinguished by distinct color coding, revealed that postprandial change in non-esterified sEH-derived diols was a strong differentiator between pro- and anti-atherogenic subjects. Specifically, 9,10-DiHOME, 14,15-dihydroxy eicosatrienoic acid (14,15-DiHETrE), and 19,20-DiHDoPE contributed maximally to the discrimination of pro- and anti-atherogenic subjects in the analysis (Fig. 4). LOX-derived metabolites also contributed to the discrimination of subjects, including non-esterified alcohols such as 5-hydroxy eicosapentaenoic acid (5-HEPE), 9-HOTE, and 9-HODE. This analysis revealed an interdependence of the effect of the meal and the atherogenic phenotype of the subject on TGRL composition.

To further evaluate the involvement of these 22 oxylipins in mediating a differential response to the meal, they were grouped into 4 clusters based on similar patterns of expression. Non-esterified diols and alcohols of C18-PUFA LA and ALA in cluster 1, represented by 9-HODE, were enriched to a greater extent postprandially in pro-atherogenic TGRL. Oxylipins in clusters 2 and 3, represented by non-esterified 5,6-DiHETrE and 19,20-DiHDoPE, respectively, consisted primarily of diols and alcohols of C20- and C22-PUFA, which were highly expressed in the fasting state in anti-atherogenic TGRL. These same metabolites were depleted in the postprandial state of anti-atherogenic subjects (Supplementary Fig. 2). In contrast, esterified diols derived from C18-PUFA LA and ALA (cluster 4), represented by 15,16-DiHODE, were enriched to a greater extent postprandially in anti-atherogenic TGRL. Consistent with the lack of inflammatory response induced by fasting TGRL in altering VCAM-1 expression, PLS-DA analysis did not discriminate pro-atherogenic from anti-atherogenic subjects when using the abundance of fatty acids and oxylipins in fasting TGRL alone. Together these data implicate heterogeneity of LOX and sEH activity in subjects producing TGRL that elicit differential inflammatory responses to the same high fat meal.

### VCAM-1 surface expression correlates strongly with the postprandial change in estimated sEH activity

A striking finding was that dyslipidemic subjects displayed a unique shift in abundance of sEH-derived diols and LOX-derived alcohols in response to the meal compared to normolipidemic subjects. We next investigated whether these subjects exhibited differences in the activity of enzymes including LOX, COX, CYP, and sEH that could account for the observed range in *ex vivo* VCAM-1 response. Indices of enzyme activity were calculated as a ratio of abundance of metabolite to parent fatty acid or precursor metabolite, independently for the esterified and non-esterified pools in TGRL. Overall the postprandial change (postprandial/ fasting) in non-esterified sEH activity index, i.e. the diol to epoxide ratio summed over all parent fatty acids measured in the non-esterified pool, strongly correlated with induction in VCAM-1 surface expression (Spearman ρ = 0.66 p = 0.04, Table 4). This enhanced activity is consistent with the PLS-DA analysis that identified the change in diols as a key feature in discriminating pro- and anti-atherogenic TGRL. Also noteworthy in the non-esterified pool was sEH activity associated with ALA, and LOX activity specific to EPA metabolism, both of which correlated strongly with pro-atherogenic activity. In the esterified pool, LOX activity linked to ALA, and CYP activity associated with EPA metabolism, were positively correlated with a pro-TGRL response. Together these results implicate a possible role for altered sEH and LOX activity in subjects whose TGRL elicits the greatest upregulation in VCAM-1 expression in inflamed HAEC.

**Table 4:** Correlation of VCAM-1 surface expression with indices of enzyme activity (n = 10).

Enzyme activity index^a^	Spearman’s ρ	P-value
**Non-esterified**		
Overall sEH activity	0.66	0.04*
• sEH activity (ALA)	0.83	0.003*
Overall LOX activity	0.24	0.51
• LOX activity (EPA)	0.72	0.02*
Overall CYP activity	−0.32	0.36
Overall COX activity	−0.21	0.56
**Esterified**		
Overall sEH activity	−0.43	0.22
Overall LOX activity	0.49	0.15
• LOX activity (ALA)	0.70	0.03*
Overall CYP activity	−0.27	0.45
• CYP activity (EPA)	0.67	0.03*
Overall COX activity	−0.41	0.24

^a^Enzymatic and lipogenesis activity were estimated using TGRL (postprandial/fasting) fatty acid and oxylipin product/ precursor relationships, separately for esterified and non-esterified pools.

^*^Significant difference.

### EPA-derived metabolites and esterified diols are predictive of changes in VCAM-1 surface expression

**Figure 5:**
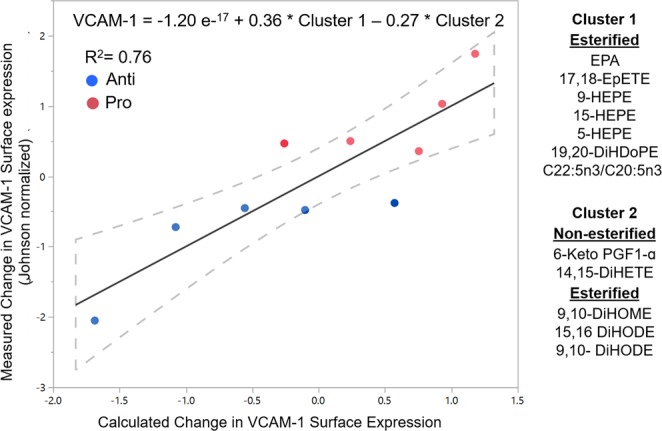
Postprandial fatty acid and oxylipin abundance in TGRL predicts changes in VCAM-1 surface expression by HAEC. The model was generated iteratively from all oxylipin, fatty acids, and calculated lipogenesis indices, clustered based on postprandial expression levels in TGRL across all subjects. Cluster scores were calculated as a linear combination of abundance of each cluster constituent, and used to fit the measured Johnson normalized VCAM-1 expression. The strongest model for predicting VCAM-1 expression consisted of a combination of two clusters as depicted. Dashed lines = 95% confidence interval, =Pro-atherogenic TGRL and =Anti-atherogenic TGRL; n = 5.

We next investigated the extent to which the abundance of specific oxylipins and fatty acids composing postprandial TGRL were predictive of the elicited changes in *ex vivo* VCAM-1 surface expression. A multiple linear regression model was generated iteratively using 19 clusters that were comprised of all the oxylipins and fatty acids and exhibited similar pattern of expression in *postprandial* TGRL across all subjects. The model with the lowest Akaike’s information criteria (AIC) score was selected as the best predictor of the postprandial VCAM-1 surface expression (Supplementary Table S4). The strongest model (R^2^ = 0.76, p-value = 0.0067) was composed of the two cluster components depicted by the equation in Fig. 5. The strongest positive correlator with the measured *ex vivo* VCAM-1 surface expression was Cluster 1, which consisted predominantly of EPA and esterified EPA-derived metabolites (17,18-EpETE, 9-HEPE, 15-HEPE, 5-HEPE). This is in accordance with the elevated EPA-specific LOX activity observed postprandially in pro-atherogenic subjects. Cluster 2, comprised predominantly of C18-derived esterified diols (12,13-DiHOME, 9,10- DiHOME, 15,16-DiHODE, 9,10-DiHODE), negatively correlated with VCAM-1 expression. These findings are consistent with the observations from the PLS-DA model.

### Reduction in lipogenesis indices and non-esterified diols in fasting subjects are strong predictors of postprandial VCAM-1 expression

**Figure 6:**
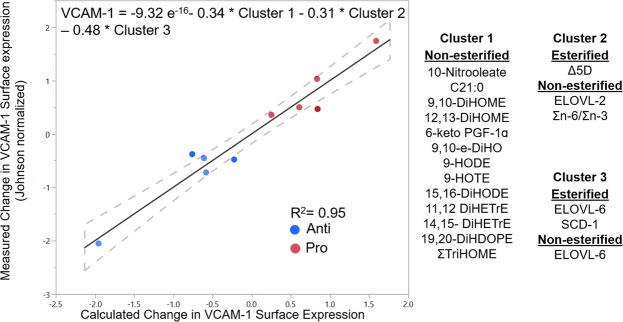
Fasting fatty acid and oxylipin abundance in TGRL predicts changes in VCAM-1 surface expression by HAEC. The model was generated iteratively from all oxylipin, fatty acids, and calculated lipogenesis indices, clustered based on fasting expression levels in TGRL across all subjects. Cluster scores were calculated as a linear combination of abundance of each cluster constituent, and used to fit the measured Johnson normalized VCAM-1 expression. The strongest model for predicting VCAM-1 expression consisted of a combination of three clusters as depicted. Dashed lines = 95% confidence interval, =Pro-atherogenic TGRL and =Anti-atherogenic TGRL; n = 5.

Given the strong correlation of postprandial VCAM-1 expression with fasting markers of dyslipidemia, we evaluated whether the composition of TGRL in fasting subjects was also predictive of the postprandial VCAM-1 response. A second multiple linear regression model was generated iteratively using 18 clusters of oxylipins and fatty acids, identified by their similar pattern of expression in TGRL from *fasting* subjects. As above the AIC score was used to select a model that best fit measured VCAM-1 expression data (Supplementary Table S5). Three clusters emerged as strong negative correlators with VCAM-1 surface expression, which provided the best fit for the data (Fig. 6, R^2^ = 0.95, p = 0.0002). Cluster 1 consisted predominantly of non-esterified diols suggesting elevated levels of sEH activity that contributes to the non-esterified oxylipin pool in fasting anti-atherogenic subjects. Clusters 2 and 3 were comprised of various lipogenesis indices and Σn-6/Σn-3, which are emerging biomarkers for dyslipidemia^28^. Both the fasting esterified and non-esterified ELOVL-6 indices (stearic acid to palmitic acid ratio) inversely correlated with VCAM-1 expression. Esterified Δ5D and SCD-1 and non-esterified levels of Σn-6/Σn-3 and ELOVL-2 were also inversely correlated to the measured VCAM-1 expression. Together, these data support the utility of these lipogenesis indices as potential biomarkers that predict a subject’s susceptibility to CVD. Moreover, the regression analysis reveals that fasting indicators of metabolism reflected in the composition of TGRL strongly predict the inflammatory postprandial response.

### Linoleic acid-derived oxylipins are regulators of TNFα-stimulated VCAM-1 expression in HAEC

**Figure 7:**
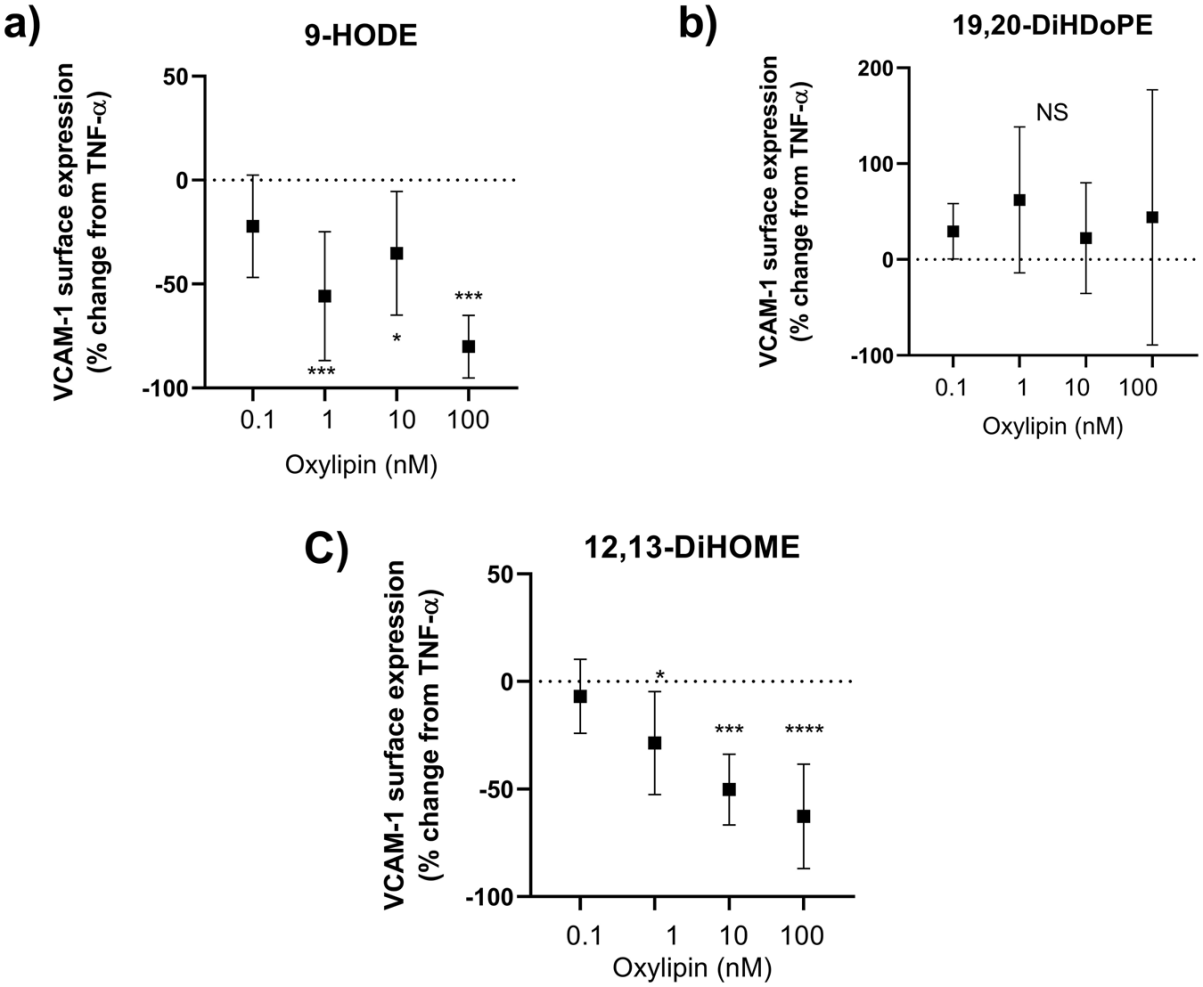
9-HODE and 12,13-DiHOME reduce VCAM-1 surface expression in TNFα-stimulated HAEC. Representative methyl esters of oxylipins identified as contributing significantly to distinguishing a pro- atherogenic phenotype were delivered to TNFα-stimulated HAEC (0.3 ng/ml, 4hrs). 9-HODE and 12,13-DiHOME reduced VCAM-1 surface expression, whereas 19, 20-DiHDoPE did not alter VCAM-1 surface expression relative to TNFα. *p < 0.05, **p < 0.005, ***p < 0.0005, ****p < 0.0001 by one-way ANOVA followed by Dunnet’s post-test. n = 3–4.

To demonstrate that the differences in concentrations of individual oxylipins identified by our analysis of TGRL could directly affect endothelial inflammation, we delivered several of them as methyl esters to stimulated HAEC and measured the dose response in VCAM-1 expression. The methyl ester form is readily taken up by cells and is analogous to the non-esterified or free form measured in TGRL. In this analysis we selected representative non-esterified oxylipins that contributed significantly to distinguishing pro- from anti-atherogenic TGRL (Fig. 4), and strongly influenced the modeled VCAM-1 expression (Figs 5 and 6). The linoleic acid metabolites 9-HODE and 12,13-DiHOME, which were present in relatively high abundance in TGRL and had a strong negative influence on the modeled VCAM-1 expression, exhibited a dose-dependent effect in reducing HAEC VCAM-1 expression (Fig. 7). On the other hand, the DHA metabolite19,20-DiHDoPE, which was present at lower abundance but was also a strong contributor to distinguishing pro-atherogenic TGRL (Fig. 4), did not itself elicit a detectable effect on VCAM-1. These data provide evidence that changes in individual oxylipins transported in TGRL could skew the nature of an inflammatory response defining an atherogenic phenotype.

## Discussion

Herein we quantify the effects of metabolic stress associated with the progression of atherosclerosis, through direct measurement of relevant inflammatory outcomes in a monolayer of arterial endothelial cells under conditions that mimic a postprandial response^15^. Our platform provided a means to directly link changes in the fatty acid and oxylipin composition of an individual’s TGRL with its inflammatory capacity measured *ex-vivo* for the first time. By characterizing TGRL before and after administration of a test meal representative of a high fat western diet, we have established a gauge for an individual’s acute postprandial inflammatory response^15–18^. Postprandial TGRL mediated a pro- or anti-inflammatory response unique to each subject that was marked by an increase or decrease in endothelial VCAM-1 receptors. The magnitude of this inflammatory response was strongly influenced by an individual’s fasting metabolic status, in that it correlated strongly with plasma markers of metabolic dysregulation and dyslipidemia. In response to the high fat meal challenge, TGRL characterized as pro- or anti-atherogenic revealed different oxylipin signatures, a key distinguishing feature of which was a shift in the relative abundance of non-esterified (free) diols. Pro-inflammatory TGRL was enriched in EPA and EPA-derived oxylipins and depleted in esterified (bound) C18-PUFA-derived diols in response to the meal, reflecting a putative shift in sEH and LOX activity in those subjects. A remarkable finding was the ability to model the measured postprandial VCAM-1 response using the relative abundance of free diols and lipogenesis indices measured in the fasting state. We conclude that specific changes in the composition of TGRL elicited by a high fat meal reflect an individual’s *in vivo* lipoprotein metabolism and mitigate the TGRL-induced *ex vivo* inflammatory response.

In quantifying the abundance of fatty acids of various saturation levels in TGRL we found that pro-atherogenic TGRL was enriched in long chain PUFA, consistent with our previous report^16^. Though PUFA are not the most abundant type of fatty acid in TGRL, by virtue of their double bonds they are readily metabolized to form oxylipins, which are known to elicit potent effects on inflammation, depending upon the metabolizing enzyme, the position of the double bond, and the parent fatty acid. Moreover, the fatty acids and oxylipins are present in both esterified and non-esterified forms in lipoproteins, which could lead to differences in their processing and effects on inflammation^10,21^. Esterified fatty acids and oxylipins in lipoproteins are released into tissues after their uptake and lipolysis^21^. We previously reported that the inflammatory response to postprandial TGRL was largely dependent upon their receptor-mediated uptake by HAEC^18^. In contrast, the non-esterified fatty acids constitute only a small proportion of the total fatty acid constituents in TGRL. However, free oxylipins and fatty acids in TGRL are more readily available upon uptake and thus may be significant mediators of endothelial inflammation. Our data points to the enrichment of non-esterified PUFA and oxylipins in pro-atherogenic TGRL, consistent with our previous analysis^16^. We proposed that the oxylipin metabolites constituting TGRL are bioactive and can in part account for the relative atherogenicity of these particles by altering endothelial inflammation.

We found that an increase in the abundance of certain vicinal (1,2)-diols, including non-esterified 9,10-DiHOME, 12,13-DiHOME, and esterified 19,20-DiHDoPE, were among the strongest discriminators of a pro-inflammatory response to postprandial TGRL. Diols are downstream products of epoxides enzymatically converted by sEH^10^. sEH inhibition is associated with anti-inflammatory and vascular relaxation properties mediated by elevated epoxides such as EETs and EpOMEs^29^. For example, 11,12-EpETrE reduced TNFɑ-induced VCAM-1 surface expression in HAEC^30^. Treatment with sEH inhibitors has also been reported to lower systemic inflammation in a mouse model of septic shock^31^, and to reduce atherosclerosis in ApoE null mice^32^. Another study observed an increase in sEH-derived diols and LOX-derived HODEs in the atherosclerotic plaques of rabbits^33^. In the current study, we observed enhanced sEH and LOX activity in dyslipidemic subjects after the high fat meal challenge. However, a higher relative abundance of non-esterified diols of C20- and C22-PUFA which were depleted postprandially was a defining feature of fasting anti-atherogenic TGRL. Our analysis consistently revealed a shift in the levels of diols in TGRL as markers of an endothelial inflammatory response. However, additional studies are needed to determine whether these diols themselves mediate the inflammatory response in HAEC, or rather reflect a shift in metabolism away from an overall anti-inflammatory state that involves cross-talk with other pathways.

This study further revealed enrichment in C18-derived midchain alcohols in the TGRL of dyslipidemic subjects. Moreover, a significant positive correlation was observed between the calculated postprandial shift in EPA-specific LOX activity and VCAM-1 expression. Although midchain alcohols are products of LOX metabolism, they can also be derived from auto-oxidation processes associated with oxidative stress^20^. As we did not directly measure the hepatic activity, the elevated LOX index may also reflect auto-oxidation within subjects. However, in monitoring markers of auto-oxidation within our samples, we found them to be expressed at low levels, and to not differ significantly between the pro- and anti-atherogenic groups. The postprandial abundance of esterified EPA and EPA-derived oxylipins (including midchain alcohols) were positive predictors for TGRL-induced VCAM-1 expression by HAEC. Additional studies are needed to determine whether the observed postprandial enrichment of EPA-derived oxylipins that mark pro-TGRL are themselves proactive mediators of inflammation.

Establishing causation for a single oxylipin in the context of a shifting oxylipin profile in the lipoprotein pools of any individual is not trivial. Indeed, an advantage of our approach is the demonstration of the functional importance of an individual’s mixture of oxylipins and their potential capacity to skew the inflammatory response. Since cells *in vivo* are exposed to a suite of fatty acids, oxylipins, and other metabolites present in lipoproteins which could have opposing effects on inflammation, characterizing the integrated response to the native oxylipin profile and evaluating correlative trends with inflammatory response *ex-vivo* provides physiological relevance. Nevertheless, herein we demonstrate that individual oxylipins identified by our analysis of TGRL could directly affect endothelial inflammation (Fig. 7). Specifically, we found that several oxylipins derived from LA that contributed negatively to the modeled VCAM-1 expression in fact dose-dependently decreased VCAM-1 expression alone. On the other hand, representative oxylipins that contributed positively in distinguishing pro-atherogenic TGRL had a neutral effect when tested alone. These data support the assertion that although isolated oxylipins may elicit an effect on inflammation, their combined effects and variable abundance in TGRL are more representative of the *in vivo* influence on inflammation. One possibility is that the expression patterns we see comparing the different responses to TGRL represent more of a shift away from an overall anti-inflammatory state to a more neutral or permissive one, rather than being overtly inflammatory. Alternatively, they may reflect a feedback response to the high fat meal in hypertriglyceridemic subjects to mitigate inflammation.

Since subjects consumed an identical high fat meal, differences in the postprandial response, especially as they correlate with markers of *fasting* metabolism, are likely attributable to altered liver metabolism. Though we did not directly measure hepatic enzyme activities as markers of liver function, elevated CRP levels have been associated with higher alkaline phosphatase and alanine aminotransferase activities in metabolic syndrome subjects. The magnitude of elevated CRP observed at fasting and postprandial in pro-atherogenic subjects could therefore indirectly point to elevated liver function^26,27^. A finding of the current study was that fasting measures of lipogenesis activity (SCD-1, ELOVL-6, and Δ5D) inversely correlated with the measured endothelial VCAM-1 expression. The liver metabolizes LA and ALA derived from the diet to produce downstream long chain PUFAs using ELOVL and desaturase enzymes. These enzymes also catalyze the production of MUFAs from SFA, and elongate SFAs. Impairment or hyperactivity of these enzymes is associated with altered lipid metabolism and disease states such as type 2 diabetes and hypertriglyceridemia, both risk factors for CVD^34–36^. We observed an overall reduction in ELOVL-6 activity in response to the high fat meal challenge, particularly in pro-atherogenic subjects, which implies impairment of elongation or saturation of palmitic acid. Elevated levels of palmitic acid are strongly associated with a sedentary lifestyle and hypertriglyceridemia^37^. In a recent ten-year longitudinal study, ELOVL-6 and Δ5D activity were significantly reduced in obese subjects who developed dyslipidemia^28^. Other reports correlated impaired Δ5D activity to high BMI, CRP, and elevated triglyceride and CVD related mortality^34,38^. Consistent with these studies, we report that Δ5D and ELOVL-6 activity inversely correlated with endothelial VCAM-1 surface expression, a direct measure of inflammation and marker of atherosclerosis^14^. These findings support the utility of measures of lipogenesis and lipid metabolism (sEH, LOX, Δ5D, ELOVL-6) in identifying subjects in a state of metabolic dysregulation associated with greater cardiovascular risk.

We have previously reported that the upregulation of TGRL-induced VCAM-1 is in part signaled via the endoplasmic reticulum (ER) stress response that promotes increased activity of the transcription factor interferon regulatory factor 1 (IRF-1)^16,18^. This mechanism distinguishes VCAM-1 expression from ICAM-1, which is independent of both IRF-1 and ER stress. By extension the varying oxylipin constituents transported in TGRL may directly induce downstream activation or inhibition of ER stress and subsequent increase or decrease in VCAM-1 surface expression. It is established that an excessive load of fatty acids can induce a state of ER stress in hepatocytes, macrophages and adipocytes^39^. ER is the hub of lipogenesis activity in cells and ER stress correlates strongly with obesity and CVD. Inhibition or knock-down of sEH can reduce high fat diet or pharmacologically induced ER stress in mouse models^40,41^. Individual oxylipins including 9-HODEs and 12/15-HETEs induced ER stress in macrophages and adipocytes, respectively^42,43^. 12(S)-HETE was shown to directly enhance the monocyte adhesion in HAEC^44^. Further, pharmacological inhibition of LOX, COX, CYP, and sEH enzymes have consistently demonstrated that perturbing oxylipin metabolism can alter systemic inflammation. For example, knock-out of 12/15 LOX in Apo-E null mice resulted in a 95% reduction in monocyte arrest within the aorta^45^. These observations motivate a need for further investigation to establish a causative role for enrichment in individual oxylipins within TGRL in modulating ER stress responses that promote endothelial inflammation.

Herein we develop regression models to predict the relative atherogenicity of an individual’s postprandial TGRL based on the oxylipins present in these lipoproteins. The models were generated from a cohort of subjects whose TGRL elicited the greatest modulation in VCAM-1 expression. Validation of these models in a larger independent cohort of subjects that includes patients with established metabolic syndrome and coronary artery disease, would ultimately demonstrate the predictive potential for gauging cardiovascular risk and disease progression over time. This approach could also prove useful in quantifying the effects of dietary intervention, statins, or other lipid-lowering therapies, by providing direct real-time measures of an individual’s lipid metabolism and inflammatory status.

The aim of this study was to shed light on the variable inflammatory response in individuals elicited by an identical meal rather than to compare the response to meals of different fatty acid composition. Thus, we chose a test meal that we have used to elicit a variable postprandial endothelial inflammatory response in multiple studies^15,16,18^. The meal is high in saturated fat and calories, and the cholesterol levels are above recommended intake for healthy diets. However, the meal is representative of a fast food breakfast meal not uncommon in the Western diet. Since this study identifies differences in the fatty acid composition of TGRL (particularly PUFA and PUFA-derived oxylipins) that correlate strongly with the nature of an inflammatory response, we envision future controlled dietary intervention studies that vary the fatty acid composition of the meal (e.g. replacing SFA with PUFA), or supplement with n-3 PUFA, and quantify the postprandial response.

Previous experience shows that the differential VCAM-1 expression observed in the postprandial state is not induced by fasting TGRL^15^. One possible explanation is that the fasting state is primarily anti-inflammatory among most subjects, though it can reflect a different baseline on which a postprandial insult superposes to amplify differences in the inflammatory response of an individual. Consistent with this is the observation that the postprandial increase in subjects’ plasma markers CRP and sVCAM predicted the magnitude of the inflammatory response to TGRL measured *ex vivo*. Thus, this technique has the potential to be employed to discriminate patient populations who do not present elevated levels of conventional clinical risk factors but are still at high risk for atherosclerotic CVD. It is also noteworthy that hypertriglyceridemic subjects exhibit lower clearance of TGRL particles and higher accumulation of lipoproteins. In this regard, the differences we have observed in the oxylipin composition between pro- and anti-TGRL may exacerbate an inflammatory response in endothelium exposed to these particles for a prolonged period.

In conclusion, we have identified a characteristic oxylipin signature in postprandial TGRL that distinguishes an acute pro- versus anti-inflammatory endothelial response in subjects consuming an identical high fat meal. Dyslipidemic subjects were uniquely characterized by a shift in sEH and LOX activity, and produced pro-atherogenic TGRL, postprandially enriched in EPA-metabolites and depleted in C18-PUFA-derived esterified diols. In contrast, normolipidemic subjects exhibited higher indices of fasting lipogenesis activity, and produced anti-atherogenic TGRL enriched in esterified C18-PUFA-derived diols, but depleted in non-esterified diols after the high fat meal challenge. These results implicate a role for the fatty acids and oxylipins constituting TGRL in mediating a pro- or anti-atherogenic endothelial phenotype. We further conclude that lipoproteins such as TGRL can serve as a depot for fatty acids and oxylipins, and propose that these bioactive fatty acid metabolites transported in TGRL could act remotely to directly modulate an endothelial inflammatory response.

## Methods

### Human subject recruitment and characterization

Human subjects (N = 39) were recruited according to an Institutional Review Board approved protocol at the University of California, Davis in accordance with the Helsinki principles and institutional guidelines and regulations. Informed consent was obtained from all research participants. The subjects varied from normal lipidemic to dyslipidemic, but did not have hypertension or elevated fasting glucose, and thus were not overtly characterized as having metabolic syndrome (Supplementary Table S1). Subjects were recruited without restriction to age, gender, race, or socioeconomic status. Exclusion criteria include subjects with kidney disease, liver disease, untreated thyroid dysfunction and ongoing hormone replacement therapy, omega-3 (n-3) fatty acid (i.e. fish oil) supplementation, lipid lowering medications (i.e. statins), excessive or prolonged use of alcohol, and consumption of nonsteroidal anti-inflammatory drugs or caffeinated beverages within 12 hrs of blood collection. Venipuncture was performed to collect 20 ml of blood after an overnight fast and again 3.5 hrs after consuming a test meal. The meal was a standardized fast food breakfast high in saturated fatty acid content and calories (Supplementary Table S3). Blood samples were immediately centrifuged (1932g, 10 min, 25 C) to obtain plasma. Plasma aliquots (both fasting and postprandial) were sent to the UC Davis Medical Center clinical laboratory for standard lipid panels, including triglycerides (TG), total cholesterol (TC), and high-density lipoprotein cholesterol (HDL). Low density lipoprotein cholesterol (LDL) was calculated using Friedewald equation. In some cases, LDL was not reported as the postprandial spike in triglycerides rendered the equation unusable. Plasma glucose and ApoB levels were also measured. A panel of cytokines and other inflammatory markers in plasma, including interleukin (IL)-1α, IL-1β, IL-6, IL-10, TNFα, IL-17A, soluble VCAM-1 (sVCAM-1), soluble ICAM-1 (sICAM-1), c-reactive protein (CRP) and interferon γ (IFNγ), were measured using a multiplex immune assay (V-Plex Meso Scale Discovery).

### TGRL isolation

Plasma was spiked with 1x antibiotic/ antimycotic (GIBCO). Density-based ultracentrifugation (40,000 RPM, 18hrs, 4 C) was used to isolate TGRL particles from plasma (ρ < 1.0063 g/ml) using a Beckman XL-90 ultracentrifuge. ApoB content was quantified using an ELISA kit (ALERCHEK) that measures both ApoB48 and ApoB100. In the fasting state TGRL consists of mainly ApoB100-containing very low-density lipoproteins (VLDL), whereas the postprandial TGRL is a mixture of VLDL and ApoB48-containing chylomicrons. ApoB concentrations were used to normalize the TGRL quantity for inflammatory characterization of HAEC and for mass spectrometry analysis. TGRL samples were characterized within 7 days of isolation or were aliquoted and frozen for future use. All samples were immediately flushed with nitrogen gas upon collection to prevent auto-oxidation. The extent of oxidation in the samples was monitored by tracking concentrations of 9-HETE, total TriHOME and F2-isoprostanes, which did not differ significantly between experimental groups.

### TGRL characterization

Human aortic endothelial cells (HAEC), passage 6–8 (Genlantis, lot #2228 derived from a 21-yr-old female, and lot #7F4409 from a 34-yr-old male), were cultured with endothelial cell growth media-2 (EGM-2, Lonza) supplemented with 1x antibiotic/ antimycotic (GIBCO). Cells were seeded on tissue culture plates (Falcon) and proliferated to form a 85–95% confluent monolayer. HAEC were then incubated with postprandial TGRL (10 mg/dl ApoB) for 4 hrs in the presence of TNFα (0.3 ng/ml, the calibrated EC_50_ for CAM upregulation, R&D). HAEC were dissociated from the tissue culture plate using 1 mM EDTA (10 mins, Sigma), and stained with phycoerythrin-conjugated VCAM-1 antibody (BD Biosciences no. 555647, 10 µl per 100,000 cells) and Alexafluor 488-conjugated intercellular adhesion molecule-1 (ICAM-1) antibody (Biolegend no. 322714, 5 µl per 100,000 cells). VCAM-1 and ICAM-1 surface expression were measured using flow cytometry (Attune NXT). To account for day-to-day cell variability in HAEC response, CAM expression is reported as % change from TNFα stimulation alone. Based on previous studies, TGRL eliciting an increase in VCAM-1 expression ≥10% are characterized as pro-atherogenic, and those that decrease VCAM-1 by ≥10% as anti-atherogenic, as these changes correlate strongly with mononuclear cell recruitment.

### Quantification of fatty acids and oxylipins in fasting and postprandial TGRL

Non-esterified fatty acids (NEFA) and oxylipins, along with total fatty acids (TFA) and alkaline stable oxylipins were quantified using mass spectrometry: ultra-performance liquid chromatography mass spectrometry (UPLC-MS/MS) for oxylipins, and gas chromatography-mass spectrometry (GC-MS) for FA. For simplicity, since the non-esterified pools account for a minor component of the total pool, the total fatty acid and total alkaline stable oxylipin pools are referred to as esterified lipids for the remainder of this manuscript. The abundance of oxylipins was reported as pmol/mg ApoB and fatty acids as nmol/mg ApoB. Lipids were isolated using a liquid-liquid extraction with cyclohexane/isopropanol/ammonium acetate after enrichment with a suite of deuterated and rare compounds used as analytical surrogates^46,47^. Fatty acids were transformed into methyl esters, using either TMS-diazomethane or methanolic transesterification for NEFA and TFA, respectively. The fatty acid methyl esters were separated on 30 m x 0.25 mm, 0.25 µm DB-225ms (Agilent Technologies), detected with a 5973 A mass selective detector (Agilent Technologies) using electron impact ionization and selected ion monitoring, and quantified against authentic standards. Results were corrected for the recoveries of analytical surrogates as previously described^47^. Non-esterified oxylipins were isolated directly from TGRL solution in PBS using solid phase extraction. Esterified oxylipins were transformed into oxylipin free acids by base hydrolysis, and isolated by subsequent hydrophilic/lipophilic interaction solid phase extraction. Non-esterified and alkaline stable esterified oxylipins were analyzed by UPLC-MS/MS using negative mode electrospray ionization and multi-reaction monitoring on a Sciex 4000 QTRAP^47^. Concentrations were calibrated using analytical standards as previously reported^47,48^.

### Characterization of oxylipin response

Oxylipins were synthesized in methyl esterified forms dissolved in ethanol by the Hammock Lab (UC Davis). They were delivered to TNFα (0.3 ng/ml) stimulated HAEC at doses ranging from 0.1–100 nM for 4hrs. Change in VCAM-1 surface expression in HAEC from vehicle control was quantified by flow cytometry.

### Enzyme activity index estimation

Fatty acid and oxylipin data were categorized into non-esterified and esterified fatty acid pools and metabolites were grouped by their parent fatty acids. An activity index for each parent fatty acid was calculated based on product to substrate abundance ratios according to the following equations:

1 $${{\rm{L}}{\rm{O}}{\rm{X}}}_{{\rm{F}}{\rm{A}}}{\rm{i}}{\rm{n}}{\rm{d}}{\rm{e}}{\rm{x}}={{\rm{\Sigma }}}_{{\rm{F}}{\rm{A}}}({\rm{m}}{\rm{i}}{\rm{d}}{\rm{c}}{\rm{h}}{\rm{a}}{\rm{i}}{\rm{n}}\,{\rm{a}}{\rm{l}}{\rm{c}}{\rm{o}}{\rm{h}}{\rm{o}}{\rm{l}}{\rm{s}}\,+\,{\rm{k}}{\rm{e}}{\rm{t}}{\rm{o}}{\rm{n}}{\rm{e}}{\rm{s}})/{\rm{p}}{\rm{a}}{\rm{r}}{\rm{e}}{\rm{n}}{\rm{t}}\,{\rm{f}}{\rm{a}}{\rm{t}}{\rm{t}}{\rm{y}}\,{\rm{a}}{\rm{c}}{\rm{i}}{\rm{d}}$$

2 $${{\rm{CYP}}}_{{\rm{FA}}}{\rm{index}}={{\rm{\Sigma }}}_{{\rm{FA}}}({\rm{epoxides}})/{\rm{parent}}\,{\rm{fatty}}\,{\rm{acid}}$$

3 $${{\rm{s}}{\rm{E}}{\rm{H}}}_{{\rm{F}}{\rm{A}}}{\rm{i}}{\rm{n}}{\rm{d}}{\rm{e}}{\rm{x}}={{\rm{\Sigma }}}_{{\rm{F}}{\rm{A}}}({\rm{d}}{\rm{i}}{\rm{o}}{\rm{l}}{\rm{s}})/{{\rm{\Sigma }}}_{{\rm{F}}{\rm{A}}}({\rm{e}}{\rm{p}}{\rm{o}}{\rm{x}}{\rm{i}}{\rm{d}}{\rm{e}}{\rm{s}})$$

An overall activity index was also estimated using the overall product to substrate ratio summed across all measured fatty acids. Analyses were then conducted on the postprandial to fasting ratio of these calculated enzyme activities.

Indices of lipogenesis were calculated as a ratio of the measured abundance of fatty acid product to precursor in the fasting and postprandial plasma or TGRL: stearoyl-CoA desaturase-1 (SCD-1, C16:1n-7/C16:0), SCD-C18 (C18:1n-9/C18:0), SCD-C16 (C16:1n-9/C16:0), delta 5 desaturase (Δ5D, C20:4n-6/C20:3n-6), elongation of very long fatty acid-6 (ELOVL-6, C18:0/C16:0), ELOVL-2 (C22:5n-3/C20:5n-3). The ratio of n-6 to n-3 PUFA was calculated across all measured PUFA (Σn-6/Σn-3).

### Statistical analysis

All statistical analyses were performed using JMP Pro 13 software. Fatty acid and oxylipin abundance data were Johnson transformed and checked for normality using the Shapiro-Wilk test. Outliers were assessed based on an interquartile range calculated per metabolite. Outliers were excluded if interquartile range <Q1 – (3 x interquartile range) or >Q3 + (3 x interquartile range). Missing values were imputed via a multivariate approach. Undetectable levels of metabolites were substituted by 0.5 x the lowest detected expression level measured for that specific metabolite.

To assess differences in FA and oxylipin composition in TGRL as a function of the meal, TGRL-induced inflammatory phenotype, and interaction between the two factors, a two-factor analysis of variance (ANOVA) was performed. P-values were corrected for multiple testing using a Benjamini-Hochberg false discovery rate (FDR) approach. Likewise, a two-factor ANOVA and FDR was used to assess differences in plasma inflammatory markers.

The Pearson method was used to correlate the change in VCAM-1 surface expression with clinical biomarkers in the full subject cohort (N = 39), since normality was satisfied. In the smaller set of subjects chosen for metabolomics analysis (n = 10), a non-parametric Spearman rank method was used to assess correlations between CAM expression and subject characteristics, plasma lipid, plasma cytokines or TGRL constituents.

To identify the shift in fatty acid and oxylipin constituents differentiating pro- and anti-atherogenic TGRL, a partial least squares discriminant analysis (PLS-DA) was performed. Postprandial abundance data were adjusted to the fasting values (postprandial/ fasting) for each subject (n = 10). This approach reduced the multivariate data for projection on the 2 coordinate axes resulting in the maximum separation between the pro- and anti-TGRL groups based on fatty acid and oxylipin abundance. The scores plot depicts the separation between the samples characterized for their relative atherogenicity, while the loadings plot superposes the metabolites that contribute to the maximum variability between the two groups. Variable importance in projection (VIP) scores the relative contribution of each metabolite to the separation visualized in the scores plot. Metabolites with a VIP score of over 1.1 were considered to contribute significantly to the separation. The leave-one-out method was used to cross-validate the model. Next, the 22 oxylipins identified as the significant contributors to the PLS-DA model were clustered by expression pattern using the JMP 13 Pro cluster variable algorithm. The fasting to postprandial change of the most representative metabolite in the cluster, i.e. that for which the expression correlated most strongly to the principal component of the cluster, was graphed in order to visualize the change in abundance of the metabolite in response to the meal.

### Regression modeling

A stepwise linear regression modeling approach was used to fit metabolite expression data to measured change in VCAM-1 surface expression in HAEC. Metabolites were first clustered to account for collinearity and maintain independence between the input variables in the model. A cluster variable algorithm in JMP Pro 13 was used to group metabolites based on the correlation of their expression levels across all subjects, using the fatty acids, oxylipins, and lipogenesis indices as inputs. This resulted in 19 clusters for the postprandial case, and 18 clusters for the fasting case. Each cluster was reduced to a representative cluster component^49^ that was an input into the iterative regression model. To satisfy the linearity requirement, VCAM-1 expression was Johnson transformed^50^. The regression model fit the response variable (y, VCAM-1 expression), using stepwise addition of cluster components as independent input variables (x_i_). The Akaike’s information criteria (AIC) and Beyesian information criteria (BIC) scores were used in model optimization with a goal of minimizing the number of independent terms. The addition of a term was considered to result in a stronger model if it improved the p-value and R^2^ while also reducing the AIC score by 2 or more^51,52^. The resulting model can be expressed by the following equation, where b_0_, b_1_, b_2_, b_3_, b_4_, …, b_x_ are coefficients corresponding to each input variable (cluster):

4 $${\rm{y}}={{\rm{b}}}_{0}+{{\rm{b}}}_{1}{{\rm{x}}}_{1}+{{\rm{b}}}_{2}{{\rm{x}}}_{2}+{{\rm{b}}}_{3}{{\rm{x}}}_{3+}{{\rm{b}}}_{4}{{\rm{x}}}_{4}\ldots .+{{\rm{b}}}_{{\rm{i}}}{{\rm{x}}}_{{\rm{i}}}$$

## Supplementary information

Supplementary Info

Raw data used for the analysis

## Data Availability

The raw data generated by the study are provided as a supplementary data file to accompany the manuscript submission.
