# Supplementary material for: Oxylipins in triglyceride-rich lipoproteins of dyslipidemic subjects promote endothelial inflammation following a high fat meal
Source: Sci Rep. 2019 Jun 17;9:8655. doi: 10.1038/s41598-019-45005-5 (PMC6572825; doi:10.1038/s41598-019-45005-5)
Supplement: Supplementary file 1 — Supplementary Info [file 41598_2019_45005_MOESM1_ESM.pdf]

Oxylipins in triglyceride-rich lipoproteins of dyslipidemic subjects promote endothelial inflammation following a high fat meal

Anita Rajamani, [1] Kamil Borkowski, [2] Samir Akre, [1] Andrea Fernandez, [1] John W. Newman, [2,3,4] Scott I. Simon, [1] Anthony G. Passerini.[1*]

[1] Department of Biomedical Engineering, University of California, Davis, 451 Health Sciences Dr., Davis, CA 95616

[2] West Coast Metabolomics Center, Genome Center, University of California, Davis, 451 Health Sciences Dr., Davis, CA 95616

[3] Department of Nutrition, University of California, Davis, 3135 Meyer Hall, One Shields Avenue, Davis, CA 95616

[4] Western Human Nutrition Research Center, Obesity and Metabolism Research Unit, Agricultural Research Service, United States Department of Agriculture, 430 West Health Sciences Dr., Davis, CA 95616

Supplementary Material

Supplementary Table S1: Anthropometric, lipid, and metabolic characteristics of subject pool before and after a high saturated fat meal

**Physical, Non-metabolic**			
Age (yrs)	39.6 ± 16.6		
BMI (kg/m^2)	25.8 ± 3.6		
Waist circumference (cm)	86.6 ± 10.7		
WHR	0.9 ± 0.1		
Systolic (mmHg)	114.8 ± 8.2		
Diastolic (mmHg)	72.3 ± 9.3		
**Metabolic, Non-lipid**	**Fasting**	**Postprandial**	**p-value**
Glucose (mg/dl)	86.2 ± 7.4	80.6 ± 11.1	0.0007
**Metabolic, Lipid**			
LDL (mg/dl) [1]	126.0 ± 64.4		
HDL (mg/dl)	58.4 ±17.3	55.6 ± 16.6	<0.0001
TC:HDL	3.8 ± 1.6	4.0 ± 1.7	<0.0001
TG (mg/dl)	100.4± 62.0	205.0 ± 139.4	<0.0001
ApoB (mg/dl)	91.3 ± 41.3	91.2 ± 41.1	0.721
Non-HDL Chol (mg/dl)	148.8 ± 69.4	149.3 ± 70.7	0.43
TC (mg/dl)	207.1 ± 69.7	204.8 ± 70.8	0.43
TG:HDL	2.1 ± 1.9	4.4 ± 3.8	0.001

Mean ± SD, N=39 (26 male and 13 female). P-values for fasting vs. postprandial comparison performed using Wilcoxon signed rank test. [1] Postprandial LDL levels could not be calculated for the majority of subjects due to elevated triglycerides.

Supplementary Table S2: Anthropometric, lipid, and metabolic characteristics of subject cohort selected for metabolomics profiling before and after a high saturated fat meal

**Physical, Non-metabolic**			
Age (yrs)	43.8 ± 20.2		
BMI (kg/m^2)	27.1 ± 4.9		
Waist circumference (cm)	89.7 ± 10.7		
WHR	0.9 ± 0.1		
Systolic (mmHg)	120.3 ± 8.0		
Diastolic (mmHg)	74.4 ± 10.9		
**Metabolic, Non-lipid**	**Fasting**	**Postprandial**	**p-value**
Glucose (mg/dl)	86.7 ± 6.3	78.9 ± 10.6	0.01
**Metabolic, Lipid**			
LDL (mg/dl) [1]	153.5 ± 117.4		
HDL (mg/dl)	52.9 ± 16.8	51.4 ± 17.2	0.29
TC:HDL-C	4.75 ± 2.6	4.91 ± 2.8	0.03
TG (mg/dl)	127.9 ± 85.8	261.6 ± 209.8	0.002
ApoB (mg/dl)	107.6 ± 72.3	111.617 ± 71.1	0.43
Non-HDL-C (mg/dl)	179.2 ± 121.5	180.4 ± 124.2	0.45
TC (mg/dl)	232.1 ± 121.6	231.8 ± 124.3	0.94
TG:HDL	3.0 ± 2.9	6.179 ± 5.5	0.002

Mean ± SD, n=10. P-values for fasting vs. postprandial comparison performed using Wilcoxon signed rank test. [1] Postprandial LDL levels could not be calculated for the majority of subjects due to elevated triglycerides.

Supplementary Table S3: Composition of the test meal

**Macronutrient**	**Value**	**Daily Value (%)**
Energy (Kcal)	1198.0	59.9
Total lipid (g)	67.5	103.8
Carbohydrate (g)	106.2	35.4
Protein (g)	44.9	89.7
SFA (g)	21.1	105.3
MUFA (g)	26.0	
PUFA (g)	11.8	
Trans (g)	1.1	
Cholesterol (mg)	508.0	169.3

Supplementary Table S4: Iterative regression model for measured VCAM-1 surface expression generated using clusters of oxylipins and fatty acids, identified by their pattern of expression in *postprandial* TGRL across all subjects.

b_0 (intercept)	b_1 Cluster 1	b_2 Cluster 2	b_3 Cluster 3	R^2	AIC	BIC	P-value
4.63 e^{-18}	0.3			0.44	32.6	29.46	0.0
**-1.2 e^{-17}**	**0.36**	**-0.27**		**0.76**	**30.1**	**23.3**	**0.0067**
-1.2 e^{-18}	0.40	-0.28	0.16	0.85	34.3	20.8	0.007

n=10, AIC: Corrected Akaike information criteria score, BIC: Bayesian information criteria score. Highlighted row corresponds to selected model in text.

Supplementary Table S5: Iterative regression model for measured VCAM-1 surface expression generated using clusters of oxylipins and fatty acids, identified by their pattern of expression in *fasting* TGRL across all subjects.

b_0 (intercept)	b_1 Cluster 1	b_2 Cluster 2	b_3 Cluster 3	b_4 Cluster 4	R^2	AIC	BIC	P-value
$-7.34\ e^{-16}$	-0.23				0.54	30.55	27.46	0.02
$-8.16\ e^{-16}$	-0.29		-0.41		0.80	28.13	21.34	0.003
**$-9.32\ e^{-16}$**	**-0.34**	**-0.31**	**-0.48**		**0.95**	**23.16**	**9.67**	**0.0002**
$-8.91\ e^{-16}$	-0.35	-0.32	-0.50	0.06	0.98	29.19	3.00	0.0002

n=10, AIC: Corrected Akaike information criteria score, BIC: Bayesian information criteria score. Highlighted row corresponds to selected model in text.

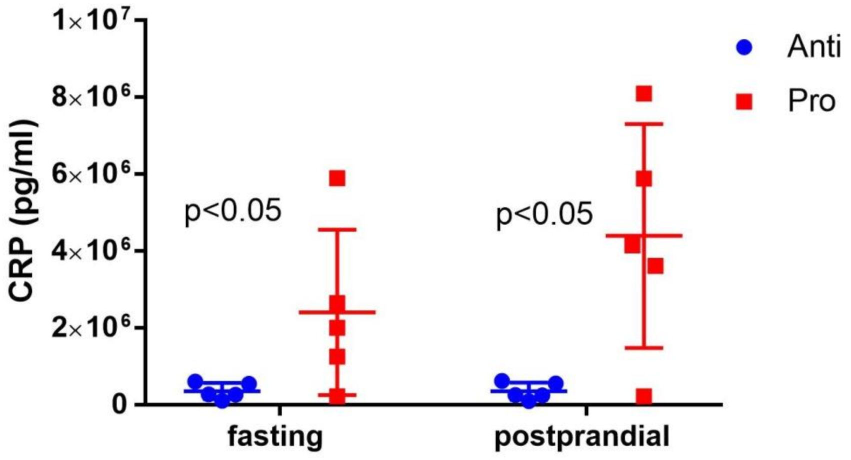

Supplementary Figure S1: Pro-atherogenic subjects are characterized by elevated plasma CRP levels at fasting and postprandial states.

An inflammatory panel measured subjects' plasma CRP levels in the fasting and postprandial states. A two-way ANOVA revealed that plasma CRP levels were significantly elevated in subjects whose TGRL were characterized as pro-atherogenic (red, n=5) compared to anti-atherogenic (blue, n=5).

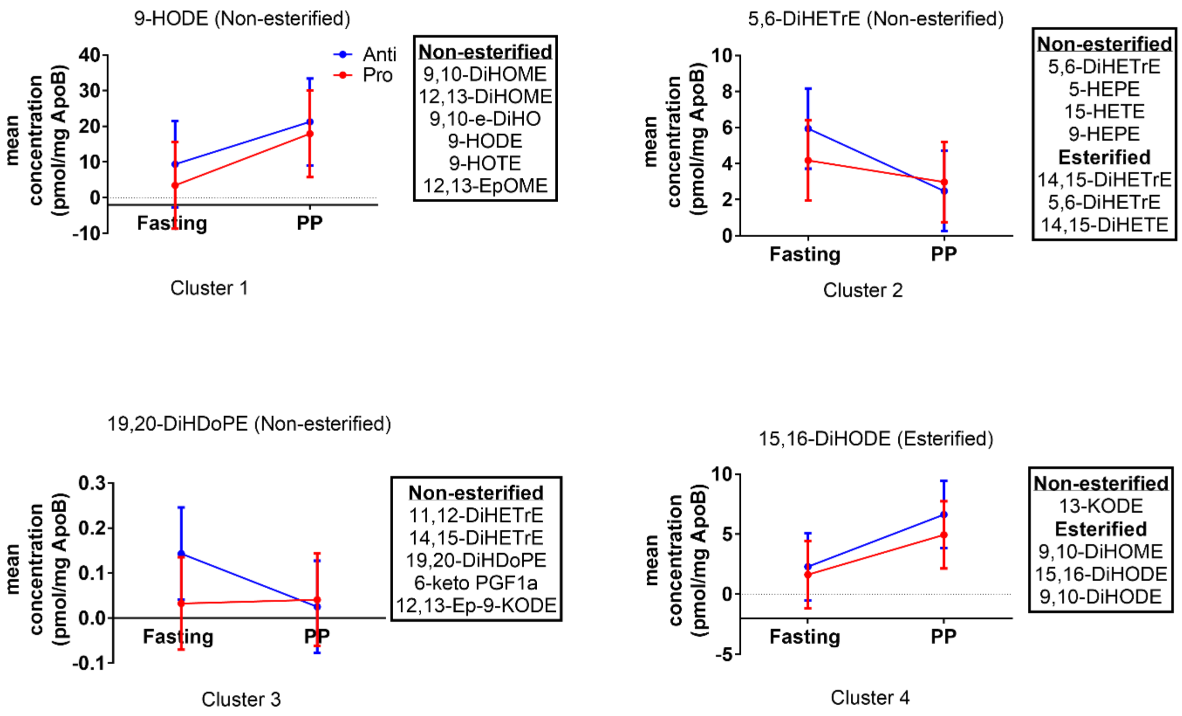

Supplementary Figure S2: Pro- and anti-atherogenic TGRL are characterized by different patterns of changing oxylipin composition in response to the high fat meal challenge.

The oxylipins identified by the PLS-DA as significant in discriminating the postprandial response between the pro- and anti- atherogenic subjects were clustered based on similar patterns of expression. The response to the high fat meal challenge is depicted for the most representative member of the clusters listed, i.e. the member that most strongly correlates with the cluster component score. Pro-atherogenic = red, anti-atherogenic = blue, n=5.
